# Accelerated food source location in aging *Drosophila*

**DOI:** 10.1111/acel.12361

**Published:** 2015-06-23

**Authors:** Sada M Egenriether, Eileen S Chow, Nathalie Krauth, Jadwiga M Giebultowicz

**Affiliations:** Department of Integrative Biology, Oregon State UniversityCorvallis, OR, 97331, USA

**Keywords:** ageing, behavior, genetics, signalling

## Abstract

Adequate energy stores are essential for survival, and sophisticated neuroendocrine mechanisms evolved to stimulate foraging in response to nutrient deprivation. Food search behavior is usually investigated in young animals, and it is not known how aging alters this behavior. To address this question in *Drosophila melanogaster*, we compared the ability to locate food by olfaction in young and old flies using a food-filled trap. As aging is associated with a decline in motor functions, learning, and memory, we expected that aged flies would take longer to enter the food trap than their young counterparts. Surprisingly, old flies located food with significantly shorter latency than young ones. Robust food search behavior was associated with significantly lower fat reserves and lower starvation resistance in old flies. Food-finding latency (FFL) was shortened in young wild-type flies that were starved until their fat was depleted but also in heterozygous *chico* mutants with reduced insulin receptor activity and higher fat deposits. Conversely, food trap entry was delayed in old flies with increased insulin signaling. Our results suggest that the difference in FFL between young and old flies is linked to age-dependent differences in metabolic status and may be mediated by reduced insulin signaling.

Fruit flies are among the best model organisms to study the biology of aging; however, their aging is most commonly assessed by lifespan, and there is a need to develop measures of changes in neural and sensory processing in aging flies. To determine whether the ability to locate food by olfaction can be used as a biomarker of aging, we compared food-finding latency (FFL) in young and old flies using a food-containing trap constructed from a microfuge tube and micropipette tip (Woodard *et al*., 1989). To examine FFL, we conducted the trap assay on young (5–10 day) and old (35–40 day posteclosion) Canton S (CS) males (see Supporting Information). To increase food search motivation (Root *et al*., 2011; Farhadian *et al*., 2012), flies were prestarved overnight (14 h) before the test. We hypothesized that functional senescence in old flies could be associated with a decreased ability to find food. Contrary to this prediction, old CS flies entered food traps in a significantly shorter time than young (Fig.1A). Likewise, old *white*^*1118*^ (*w*) flies showed significantly shorter FFL compared to young (Fig.1B).

**Fig 1:**
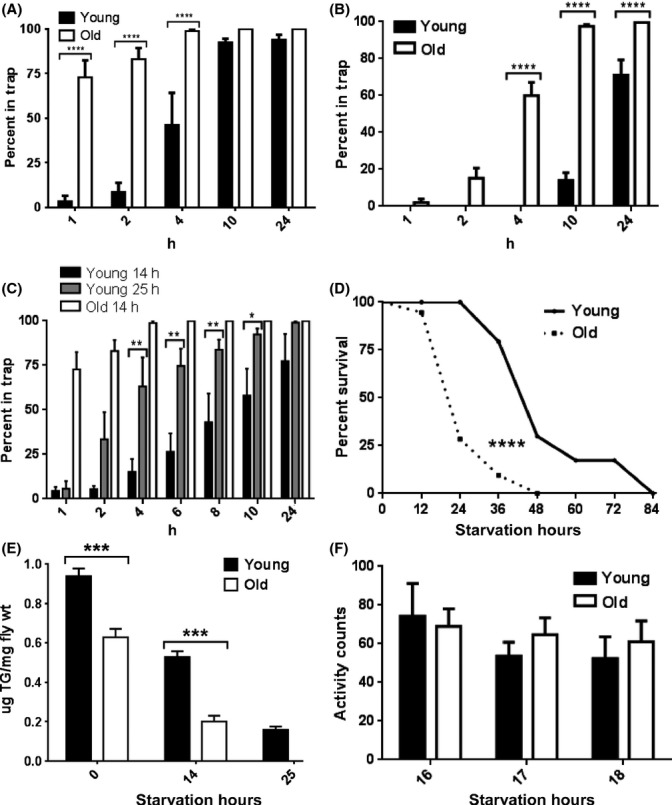
Old flies show decreased food-finding latency (FFL). (A, B) A significantly higher proportion of old CS (A) and *w* (B) flies entered food traps during the first 10 h of testing. (C) Young CS flies fasted for 25 h prior to the trap experiment show significantly decreased FFL compared to young flies fasted for 14 h, but similar FFL to old flies fasted for 14 h. Bars in A–C represent average (± SEM) percentage of flies in food trap (*N* = 6 food traps per condition with 20–25 flies in each trap). (D) Starvation resistance is significantly lower in old CS flies (median survival = 24 h) compared to young (median survival = 48 h); *N* = 75 flies per condition, survival curves analyzed by log-rank test. (E) Old CS flies have significantly lower fat levels prior to starvation and significantly reduced fat levels after 14 h of starvation. Bars show average (± SEM) TG levels based on *N* = 5 samples. (F) Average (± SEM) locomotor activity measured 16–18 h after onset of starvation was not significantly different in old and young flies (*N* = 7 for young, *N* = 11 for old). Data in A–C and E–F were analyzed by two-way ANOVA corrected for multiple comparisons with Bonferroni post-test. *P < 0.05, **P < 0.01, ***P < 0.001, ****P < 0.0001.

This reduced FFL could be caused by many factors such as an increased state of hunger in old relative to young flies. To test this, we monitored the number of flies entering food traps in young CS flies starved for 14 h or 25 h along with old flies starved for 14 h. Young flies starved for 25 h had significantly shorter FFL than young flies starved for 14 h (Fig.1C, and Supporting Movie). There was no significant difference in FFL between young flies starved for 25 h and old flies starved for 14 h, suggesting that reduced FFL is related to nutritional state. We further confirmed this by testing starvation resistance of young and old CS flies. Under complete food deprivation, median survival time was significantly shorter in old flies (24 h) than in young (48 h) (Fig.1D), which suggests that old flies have lower energy stores. To test this, we measured triglyceride (TG) levels as a function of starvation time. Steady-state TG levels were significantly lower in old than in young fed CS flies (Fig.1E), as previously suggested (Katewa *et al*., 2012). TG levels were reduced during fasting, and comparable levels were found between young flies starved for 25 h and old flies starved for 14 h (Fig.1E). These data are consistent with the difference in mean survival under starvation between young and old flies (Fig.1D) and suggest that decreased FFL in old flies is correlated with decreased fat stores.

It has been reported that food shortage increases locomotor activity in young flies and this may facilitate foraging behavior (Lee & Park, 2004; Katewa *et al*., 2012). We measured the amount of locomotor activity in young and old CS flies under food deprivation during the time when significant difference is observed in trap entries. Total activity counts were not significantly different (Fig.1F), suggesting that shorter FFL in old flies is not caused by higher overall locomotor activity. However, this conclusion is tentative as the activity measures were made on single flies in Trikinetics tubes while FFL was measured in a round arena containing 25 flies.

Food location behavior in young food-deprived flies is modulated by reduced insulin/IGF signaling (IIS) that enhances olfactory processing of food-related odors in specific olfactory neurons (Root *et al*., 2011). It is also known that fat cells of starved flies suppress the release of the insulin-like peptide from the brain causing systemic IIS reduction in flies (Geminard *et al*., 2009). Therefore, we asked whether old flies show more robust food search behavior due to lower IIS. If such was the case, young flies with reduced IIS should enter food traps faster than controls. To test this prediction, we measured FFL in mutants in the gene *chico*, which encodes the insulin receptor substrate protein. We used heterozygous *chico*^*1*^/+ flies, which were shown to have normal size and extended lifespan (Clancy *et al*., 2001). Remarkably, young *chico*^*1*^/+ flies starved for 14 h showed significantly shorter FFL than *w* controls (Fig.2A), and this was even true in old *chico*^*1*^/+ flies (Figure S1A). To dissect the role of fat reserves versus IIS in modulating FFL, we measured TG levels in young *chico*^*1*^/+ and controls starved for 14 h. Interestingly, TG levels were significantly higher in *chico*^*1*^/+ flies (Fig.2B), and these mutants also showed significantly higher starvation resistance compared to controls (Fig.2C). These data are consistent with previous reports linking reduced IIS with an increase in both TG and starvation resistance in flies (Haselton *et al*., 2010). Shorter FFL in young *chico*^*1*^/+ flies does not appear to be caused by increased locomotor activity, as average activity counts were similar between *chico*^*1*^/+ and *w*^*1118*^ controls (Fig.2D), although, again, the assay environments were different. Together, these data suggest that accelerated food finding in old flies is not controlled directly by reduced fat reserves, but rather indirectly, via lowered insulin signaling. In support of this link, we also determined that FFL was significantly extended in old flies with increased IIS via RU-induced neuronal expression of constitutively active insulin receptor (InR^CA^) in adult flies (Figure S1B). Previous studies showed that the modulation of insulin signaling in olfactory receptor neurons affects odor-driven food search behavior in young flies (Root *et al*., 2011). Our data suggest that lower fat reserves in old flies may shorten FFL in aging flies via reduced insulin signaling, which may sensitize olfaction pathways to food-related odors. While our data suggest that insulin signaling is involved, it is not yet clear whether shortened FFL in old flies is mediated by reduced insulin signaling or whether nutritional state and insulin signaling affect FFL through parallel pathways.

**Fig 2:**
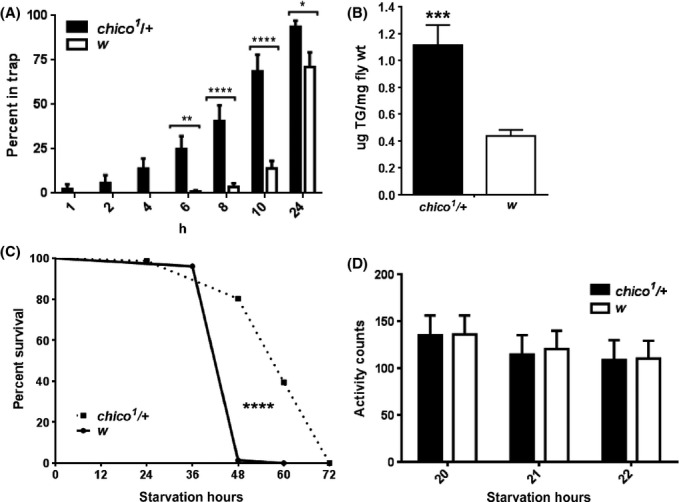
Reduced insulin signaling leads to decreased FFL in young flies despite their increased fat stores. (A) Young *chico*^*1*^*/+* flies enter traps significantly sooner than *w* control flies of the same age. Bars represent average (± SEM) based on *N* = 6 food traps per genotype. (B) Fat levels measured after 14 h of starvation are significantly higher in young *chico*^*1*^*/+* flies than in controls of the same age (*N* = 3, data analyzed by unpaired t-test). (C) Young *chico*^*1*^*/+* flies survived significantly longer under starvation than *w*. *N* = 75 flies for each genotype, survival curves analyzed by log-rank test. (D) Average locomotor activity measured 20–23 h after onset of starvation was not significantly different between young *chico*^*1*^*/+* and *w* control flies (*N* = 23 for *chico*^*1*^*/+*, *N* = 13 for *w*). Data in A and D were analyzed by two-way ANOVA corrected for multiple comparisons with Bonferroni post-test; *P < 0.05, **P < 0.01, ***P < 0.001, ****P < 0.0001. The *chico*^*1*^mutants obtained from Bloomington Drosophila Stock Center (# 10738) were backcrossed to *w*^*1118*^ flies for 6 generations and tested as heterozygotes along with *w*^*1118*^ background control.

In summary, we show here that the physiological systems facilitating the location of a food source are fully functional in old flies. While aging in flies has been linked to impaired memory and reduced avoidance of adverse odors (Tonoki & Davis, 2012), neuronal substrates and processes necessary for survival-aiding behaviors are preserved in old flies and can be recruited upon a loss of nutritional homeostasis.

## Supporting Information

**Fig. S1** (A) Old *chico*^*1*^*/+* flies enter traps significantly sooner at 1 h and 2 h after onset of food trap experiment than control flies of the same age. (B) Neuronal expression of constitutively active insulin receptor (InR^CA^) delays entry to food traps in old flies. Adult *elav-GS>UAS-InR^CA^* males were kept on diet containing 200uM of RU-486 in ethanol (RU+) or ethanol alone (RU−). Bars in A-B represent average (±SEM) percentage of flies in food traps (N=6 food traps per condition in A, N=3 in B). Data in A-B were analyzed by two-way ANOVA corrected for multiple comparisons with Bonferroni post-test; **p* < 0.05, ***p*<0.01.

**Movie S1** Time lapse video demonstrating difference in food source location young flies pre-starved for 14 h and 25 h, after 0 h, 2 h, 4 h, and 24 h in food trap arenas.
