# Supplementary material for: Accelerated food source location in aging Drosophila
Source: Aging Cell. 2015 Jun 23;14(5):916–8. doi: 10.1111/acel.12361 (PMC4568979; doi:10.1111/acel.12361)
Supplement: Supplementary file 1 [file acel0014-0916-sd1.docx]

**Supplemental Information**

**Accelerated Food Source Location in Aging *Drosophila***

Sada Egenriether, Eileen Chow, Nathalie Krauth, Jadwiga Giebultowicz

**Experimental Procedures**

**Fly rearing and genotypes**

*Drosophila melanogaster* were reared on diet containing 1% agar, 6.25% cornmeal, 6.25% molasses, and 3.5% Red Star yeast at 25 ºC. Flies were entrained to 12-hour light:dark (LD, 12:12) cycles (with an average light intensity of ~1500 lx). Genotypes used in this study were Canton S (CS), *white* (*w^1118^*), *elav-GS* ([Osterwalder *et al.* 2001](#_ENREF_3)), *UAS-InR^CA^* **(**Bloomington Drosophila Stock Center # 8250**)** and *chico^1^* (Bloomington Drosophila Stock Center # 10738). *chico^1^* mutants were backcrossed to *w^1118^* flies for 6 generations and tested as heterozygotes along with *w^1118^* background control. All experiments were performed on mated male flies of different ages, as specified in the results.

**Food trap assay**

To determine how aging affects food search behavior we used a food-filled trap constructed from a microfuge tube and micropipette tip as previously described ([Woodard *et al.* 1989](#_ENREF_4)). The lid of each microfuge tube was filled with regular food. To increase food search motivation, flies were starved overnight (14h) before the test (25h in one experiment). Flies were briefly immobilized on ice and 20-25 flies were placed in a petri dish arena (Fisher Scientific, 100 x 20mm) containing a food trap. In preliminary experiments, a second, water filled trap was included in addition to the food trap to test whether flies were entering traps for food rather than curiosity. We never observed more than one fly in the water trap at the end of the 24 hour trapping period, so this step was omitted in subsequent experiments. A moistened Kimwipe was included in each trap arena to ensure flies were entering traps in search of food, rather than water.

Flies were held in constant darkness during the test and numbers inside/outside of traps were counted at 1-2 h intervals under dim red light. Flies that died during an interval were not included in total count when calculating the percent that found food during that interval. For each genotype and age combination, 4-12 individual petri dishes were averaged for percent in trap at each time interval. To compare food search behavior between young flies fasted 14h versus 25h, the trap assay was performed as described above, with one group pre-fasted for 25h prior to the experiment, and the second group fasted for the usual 14h.

**Starvation assay**

To compare starvation resistance between old and young flies, three groups of 25 males aged 5d and 35d were loaded into vials containing 1% agar and mortality was recorded at 12 hour intervals.

**TG assay**

Triglyceride levels were measured using Stanbio Liqui-color Triglycerides kit (Stanbio labs, Boerne, TX) ([Katewa *et al.* 2012](#_ENREF_1)). The weight of 5 males was recorded, and weighed flies were subsequently homogenized in PBST and centrifuged for 1 min at 5000rpm. Aliquots of vortexed supernatant were processed for triglyceride measurement as per manufacturer instructions.

**Activity monitoring**

For measurement of spontaneous activity we used *Drosophila* activity monitors (Trikinetics Inc., Waltham, MA) as described ([Long *et al.* 2014](#_ENREF_2)). Flies were placed in tubes containing agar and activity counts were automatically recorded every 15 minutes then averaged as counts/h separately for young and old flies of different genotypes.

**Supplementary references**

Katewa SD, Demontis F, Kolipinski M, Hubbard A, Gill MS, Perrimon N, Melov S, Kapahi P (2012). Intramyocellular Fatty-Acid Metabolism Plays a Critical Role in Mediating Responses to Dietary Restriction in Drosophila melanogaster. *Cell metabolism*. 16, 97-103.

Long DM, Blake MR, Dutta S, Holbrook SD, Kotwica-Rolinska J, Kretzschmar D, Giebultowicz JM (2014). Relationships between the circadian system and Alzheimer's disease-like symptoms in Drosophila. *PLoS One*. 9, e106068.

Osterwalder T, Yoon KS, White BH, Keshishian H (2001). A conditional tissue-specific transgene expression system using inducible GAL4. *Proc Natl Acad Sci U S A*. 98, 12596-12601.

Woodard C, Huang T, Sun H, Helfand SL, Carlson J (1989). Genetic analysis of olfactory behavior in Drosophila: a new screen yields the ota mutants. *Genetics*. 123, 315-326.

**Movie S1**

Time lapse video demonstrating difference in food source location young flies pre-starved for 14h and 25h, after 0h, 2h, 4h, and 24h in food trap arenas.

(Supplemental Movie)

**Figure S1**


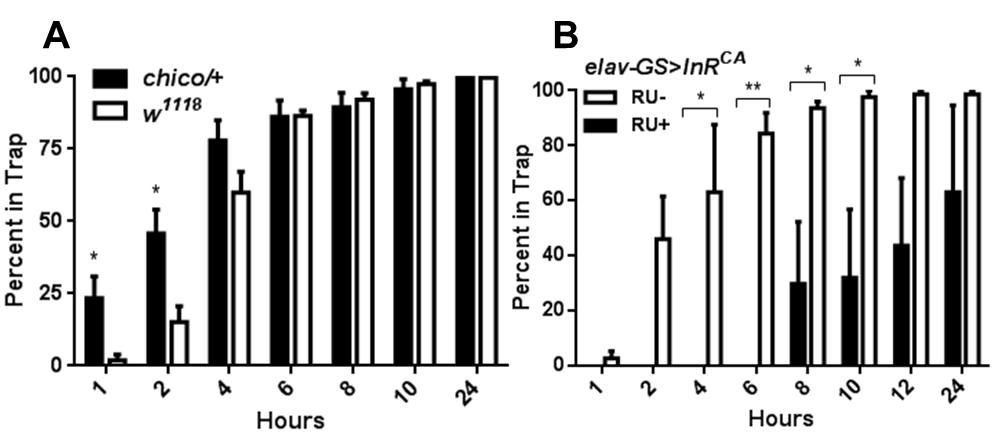


**Supplementary Figure 1**. (A) Old *chico^1^/+* flies enter traps significantly sooner at 1h and 2h after onset of food trap experiment than control flies of the same age. (B) Neuronal expression of constitutively active insulin receptor (InR^CA^) delays entry to food traps in old flies. Adult *elav-GS>UAS-InR^CA^* males were kept on diet containing 200uM of RU-486 in ethanol (RU+) or ethanol alone (RU-). Bars in A-B represent average (±SEM) percentage of flies in food traps (N=6 food traps per condition in A, N=3 in B). Data in A-B were analyzed by two-way ANOVA corrected for multiple comparisons with Bonferroni post-test; *p < 0.05, **p<0.01.
